# A Conserved Allosteric Site on Drug-Metabolizing CYPs: A Systematic Computational Assessment

**DOI:** 10.3390/ijms222413215

**Published:** 2021-12-08

**Authors:** André Fischer, Martin Smieško

**Affiliations:** Computational Pharmacy, Departement of Pharmaceutical Sciences, University of Basel, Klingelbergstrasse 61, 4056 Basel, Switzerland; and.fischer@unibas.ch

**Keywords:** Cytochrome P450, CYP, allosteric, ligand access, tunnel, molecular dynamics, bioinformatics

## Abstract

Cytochrome P450 enzymes (CYPs) are the largest group of enzymes involved in human drug metabolism. Ligand tunnels connect their active site buried at the core of the membrane-anchored protein to the surrounding solvent environment. Recently, evidence of a superficial allosteric site, here denoted as hotspot 1 (H1), involved in the regulation of ligand access in a soluble prokaryotic CYP emerged. Here, we applied multi-scale computational modeling techniques to study the conservation and functionality of this allosteric site in the nine most relevant mammalian CYPs responsible for approximately 70% of drug metabolism. In total, we systematically analyzed over 44 μs of trajectories from conventional MD, cosolvent MD, and metadynamics simulations. Our bioinformatic analysis and simulations with organic probe molecules revealed the site to be well conserved in the CYP2 family with the exception of CYP2E1. In the presence of a ligand bound to the H1 site, we could observe an enlargement of a ligand tunnel in several members of the CYP2 family. Further, we could detect the facilitation of ligand translocation by H1 interactions with statistical significance in CYP2C8 and CYP2D6, even though all other enzymes except for CYP2C19, CYP2E1, and CYP3A4 presented a similar trend. As the detailed comprehension of ligand access and egress phenomena remains one of the most relevant challenges in the field, this work contributes to its elucidation and ultimately helps in estimating the selectivity of metabolic transformations using computational techniques.

## 1. Introduction

**Figure 1:**
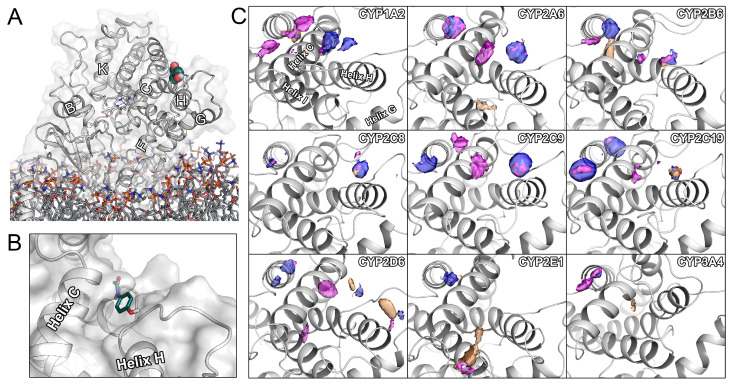
Structural overview and cosolvent densities. (**A**) Structure of membrane-anchored CYP2D6 with a ligand bound to the H1 site (shown as spheres). For orientation, helices have been denoted with letters. (**B**) Close-up view of the H1 allosteric site in CYP2D6 with bound ligand acetaminophen. (**C**) Probe densities obtained from cosolvent MD simulations. Pink densities correspond to pyridine, blue ones to isopropanol, and orange ones to acetonitrile. The probe densities are shown at an isolevel of 15 σ above the basal level of occupancy.

Cytochrome P450 enzymes (CYPs) are the most relevant class of enzymes responsible for the biotransformation of approximately 70–80% marketed of drugs. CYPs can catalyze a range of oxidative and reductive reactions including hydroxylation, heteroatom oxygenation, dealkylation, and epoxidation with a distinct substrate specificity [1,2]. Clinical complications related to metabolism can occur due to potential drug–drug interactions and interindividual differences resulting from genetic polymorphism, both altering drug elimination [1,3,4]. The prediction of CYP metabolism, both in regard to ligand selectivity and catalytic efficiency, is of pivotal importance for rational design as a large share of drug attrition is caused by a poor pharmacokinetic profile [5,6]. Even though differences in active site residues partially allow for the explaination of the complex substrate specificity of CYPs, other structural mechanisms such as ligand access and egress have been evidenced to influence it [7,8,9,10]. In particular, the active site of CYPs is buried within the core of the protein and is connected to its surrounding environment by dynamic tunnels. The most narrow region of such a tunnel is referred to as bottleneck, where gating residues act as molecular filters for compounds accessing the enzyme. The opening of these tunnels depends on conformational changes of the protein. As mammalian CYPs are membrane-anchored proteins (Figure 1A), it is thought that lipophilic ligands access the binding site through membrane-facing tunnels, while hydrophilic compounds, including the metabolic products, prefer tunnels reaching the bulk solvent [8,10,11,12]. In general, experimental methods can only provide limited insight into such ligand translocation processes [8] as, for example, the tunnels are often not apparent in static crystal structures. However, due to their dynamic nature, computational methods such as molecular dynamics (MD) simulations introducing a natural degree of structural flexibility have been widely applied to study ligand tunnels in atomic detail [8,10,13,14,15].

Allosteric regulation of enzymatic activity is a powerful mechanism for cells to adapt to their cellular environment by propagating information between two distinct sites of the protein [16,17]. Only recently, a superficial allosteric site among helices C, E, and H (Figure 1B) was proposed to be involved in the regulation of substrate access to the soluble prokaryotic enzyme CYP101A1. Upon ligand association at this site, the enzyme was described to shift from a closed to an open conformation, facilitating the access of other ligands through a tunnel located between the F-G loop and the B-C loop [14]. Especially in the presence of a high substrate concentration, allosteric regulation may allow adaption to the environment by enhancing metabolic activity, thus functioning as a protective mechanism to facilitate the excretion of large amounts of potentially toxic xenobiotics. In our recent work focused on CYP2D6 ligand access, we could verify the association of small molecules at this site, which we denoted as hotspot 1 (H1). Similar to the above-mentioned study by Follmer and colleagues, we examined ligand access and observed a considerable correlation between the occupancy of the H1 site and complete ligand translocation to the active site [8].

Intrigued from previous findings regarding the H1 site, this work was focused on elucidating its functionality in a panel of nine mammalian drug-metabolizing CYPs covering the majority of phase I metabolism. We systematically applied bioinformatic tools, cosolvent simulations, conventional MD (cMD) simulations, and metadynamics simulations to study in detail CYP1A2, CYP2A6, CYP2B6, CYP2C8, CYP2C9, CYP2C19, CYP2D6, CYP2E1, and CYP3A4 enzymes. The enzymes were selected based on their relevance for the metabolism of clinically used drugs (Table S1) [1]. We examined conservation, small-molecule association, conformational adaptation, and the facilitation of ligand translocation. Our results indicate that the site is mostly conserved among the CYP2 family. Additionally, the association of organic probes and small molecules revealed a nearby site separated from H1 by helix C. Ultimately, metadynamics simulations indicated the occupation of H1 site to facilitate ligand egress in most systems, with statistically significant differences in CYP2C8 and CYP2D6, although we did not detect a transition to an open conformation as it was described for CYP101A1. Our work improves the understanding of the allosteric regulation of ligand access to drug-metabolizing CYPs, a process closely related to their substrate specificity and relevant for the accurate prediction of metabolic outcomes.

## 2. Results and Discussion

### 2.1. Simulation Techniques and Model Validation

By introducing flexibility into molecular systems, MD simulations find many different applications such as studying time-evolved ligand–protein interactions, conformational changes in protein structures, assessing the role of solvent molecules, or identifying putative binding sites [18,19,20]. Here, we applied various MD techniques including cMD simulations, metadynamics, and cosolvent simulations to study the most relevant drug-metabolizing CYPs. By introducing molecular probes to a simulation system, one can observe their association with the protein of interest and highlight potential binding sites [18]. Based on these simulations, we could deduce different pharmacophores of the H1 site. Due to the short timescale of these simulations (Table 1) preventing them from significant structural changes, we modeled the soluble protein without the membrane. Similar to the cosolvent simulations with small organic probes, we evaluated the association of small drug-like compounds with the proteins. Next, in order to investigate the effect of allosteric ligands bound to the H1 site proposed by Follmer and colleagues [14], we placed 20 ligands around the protein and observed their association with the H1 site. The root mean-square deviation (RMSD) indicated good convergence of the respective simulations (Figure S1). As mentioned above, mammalian drug-metabolizing CYPs are membrane-anchored enzymes. The structural regions of CYPs involved in forming several well-characterized access tunnels are in direct contact with head groups of the membrane molecules [8,10,11,21]. Thus, to accurately study the opening and closing of ligand tunnels, we constructed membrane models for each enzyme by adding membrane anchors (as detailed in the Materials and Methods section) and embedding the protein in a preequilibrated 1-palmitoyl-2-oleoylphosphatidylcholine (POPC) membrane. The orientation and embedding of the protein was predicted using the well-established PPM server, similar to our previous work [8,21]. The resulting systems were subjected to a 300 ns equilibration simulation, during which we computed two metrics commonly used to characterize and validate models of membrane-anchored CYPs. The heme tilt angle is defined as the angle of the plane of the porphyrin nitrogens of the heme moiety and the membrane normal corresponding to the z-axis of the system [11,21]. Based on rotational diffusion measurements, the heme tilt angle for CYPs was determined to be in the range of 38–78${}^{\circ }$ [22]. The average values determined in the equilibration simulations ranged between 53.1 and 73.3${}^{\circ }$ in agreement with the experimentally determined boundaries (Table S2). Further, the angle remained relatively stable around the starting value, indicating that there were no large changes in respect to the input structures (Figure S2). When compared to previous results by Berka and colleagues [11], the values were highly similar for CYP1A2, CYP2C9, and CYP2E1, while there were considerable differences for CYP2A6, CYP2D6, and CYP3A4. However, when we compared the results of CYP2D6 to our previous work [8], the observed angles were again highly similar. Another parameter we monitored was the burying depth of the globular domain of each enzyme, which was experimentally determined to be 35 ± 9 Å for CYP2B4 by atomic force microscopy [23]. In our equilibration simulations, we observed burying depths between 35.7 and 38.9 Å in agreement with experimental observations. In addition to the heme tilt angle and the burying depth, we assessed the backbone RMSD of the simulations, which indicated acceptable convergence (Figure S3).

Using the validated membrane models, we conducted microsecond simulations with an allosteric ligand bound and, after 5 ns of unrestrained simulations, restrained them to the H1 site. In these simulations, we observed high RMSD values for CYP1A2, CYP2A6, CYP2C19, and CYP2D6 (Figure S4). Upon inspection of the trajectories, we could observe large structural changes of the membrane anchor, while the globular domain and the overall fold of the protein remained stable (Figure S5). In a last step, we conducted metadynamics simulations focused on studying ligand egress from the buried binding site with and without the presence of an allosteric ligand bound to H1. Due to the conformational changes imposed by the translocating ligand, one replica simulation of CYP2C8 as well as multiple simulations of CYP1A2 presented increased values compared to the remaining enzymes, which showed acceptable RMSD values below 4 Å (Figures S6 and S7). However, high values were to be expected due to the application of biasing potentials to the systems and the propagating ligand.

### 2.2. Small Molecules Regularly Associate with the H1 Site of Several CYPs

As mentioned in the previous section, cosolvent MD simulations can be applied to map binding sites of a protein by introducing small organic probes. Here, we conducted simulations with acetonitrile, isopropanol, and pyridine to compare the obtained densities among the panel of CYPs (Figure 1C). We detected considerable density of isopropanol and pyridine probes at the H1 site of all studied CYPs besides CYP2E1 and CYP3A4. In CYP2B6, CYP2C8, CYP2C19, CYP2D6, and CYP3A4, there were densities of acetonitrile, even though they were less pronounced. Interestingly, only the densities of pyridine presented an overlap between the two highly similar (regarding sequence) enzymes CYP2C9 and CYP2C19. Instead, CYP2C9 was more similar to CYP2A6 with an isolated density of of pyridine close to the center of helix C as well as a shared density of pyridine and isopropanol between the center of helix C and the C-terminal of helix H. In all enzymes except for CYP2E1 and CYP3A4, this distribution of densities was present. Besides the densities at the H1 site, we could detect the association of probes on a neighboring site, which is separated from H1 by helix C. At the given isovalue of 15, every CYP studied here presented a density of at least one probe in this region. However, this adjacent density was comparatively small in CYP2C8 and CYP2E1. In CYP2C19, the density of the neighboring site was even larger than the ones detected at the H1 site.

**Figure 2:**
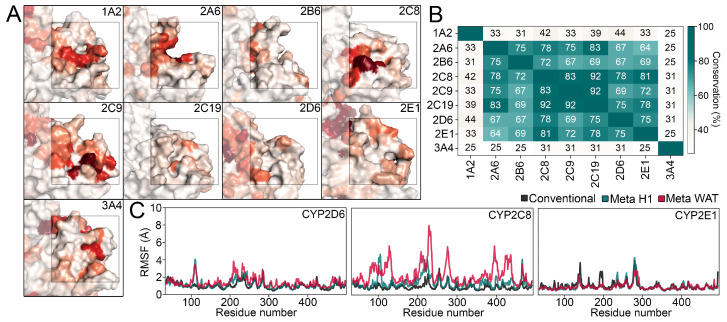
(**A**) Small-molecule binding hotspots on the surface of the nine enzymes studied in this work. Different shades of red indicate the degree of ligand association per residues as described in the Materials and Methods section. (**B**) Heatmap depicting the conservation of the H1 site within the panel of CYPs. (**C**) Analysis of structural changes observed in metdynamics simulations.

Previously, the association of the small molecules camphor and acetaminophen with the H1 site of drug-metabolizing CYPs were reported in the literature as well as our previous work [8,14]. Additional evidence for the association of small-molecules at the H1 site was provided by X-ray crystallography of CYP101A1 cocrystallized in excess of camphor. The resulting structure revealed a slight electron density of camphor at the H1 site [14,24]. Thus, we decided to study the association of drug-like molecules with the H1 site in addition to the small organic probes in cosolvent simulations. CYP1A2, CYP2A6, CYP2C8, and CYP2C9 presented a high degree of ligand association at the H1 site (Figure 2A). In analogy to the cosolvent simulations, only a minor association of ligands at the H1 site was seen at CYP2E1 and CYP3A4, indicating a missing functionality of this site in the context of regulation by small molecules. In contrast to our previous findings [8], CYP2D6 only showed limited association of ligands with the H1 site, but rather with the neighboring site separated from H1 by helix C. Generally, the hotspots roughly overlapped with the regions showing increased probe densities in cosolvent simulations.

To assess the conservation of a protein region, sequence alignments followed by the comparison of the respective residues is a commonly used technique [25]. We observed a high conservation of the site in the CYP2 family, especially among CYP2A6, CYP2B6, CYP2C8, and CYP2C19 (Figure 2B). Especially in the CYP2C subfamily, the identity among the studied members was above 83%. As it is implied by the nomenclature [2], CYP3A4 shared less sequence identity with the CYP2 family. Again, this stands in accordance with the results from the MD simulations discussed above. In contrast to the results of the small-molecule association simulations, the residues in the H1 site of CYP2E1 presented a considerable degree of conservation to other enzymes in the CYP2 family.

### 2.3. The Effect of Allosteric Ligands Bound to the H1 Site Is Isoform-Dependent

As mentioned above, Follmer and colleagues observed a shift to an open conformation in response to a ligand bound to the H1 site of CYP101A1. To potentially translate those results to mammalian CYPs, we used our membrane-anchored CYP models and restrained a ligand to the H1 site to ensure constant occupancy of the allosteric site. In the microsecond simulations, we monitored the conformational state of the enzyme, as well as the three most relevant ligand tunnels described in the literature [8,10,11,14,15,26]. These tunnels included tunnel 2b located among the B-C loop, the F-G loop, and the β4 sheet; tunnel 2f located between helix A and the F-G loop; as well as tunnel 2c between the N-terminus of helix I and the B-C loop [8,15,21]. After processing MD frames from the simulations using CAVER [27], we determined the average bottleneck radii of the three tunnels and statistically compared them to the values obtained from the final frames of the membrane equilibration simulations (Table 2, Tables S3 and S4). Whereas there was no clear trend for an enlargement of tunnel 2c, enzymes of the CYP2 family including CYP2B6, CYP2C8, CYP2C9, and CYP2C19 presented a statistically significant increase in the bottleneck radius of tunnel 2f (Figures S8–S10). In the work on CYP101A1, the F-G loop was described as a key regulatory structure for the opening of tunnels [14]. As this loop separates tunnel 2f and 2b, a slight movement away from the tunnel entrances can lead to the merging of these tunnels. In CYP2B6, we could observe an enlargement of both tunnel 2b and 2f, potentially connected to the increase in the root mean-square fluctuation (RMSF) of two replica simulations between residues 220 and 250 (Figure S11). Indeed, the other enzymes of the CYP2 family, sharing an increased opening of tunnel 2f if an allosteric ligand was present, exhibited a similar behavior in the RMSF diagrams. Thus, rearrangements of the helices F and G, as well as the loop connecting them, are likely responsible for the observed behavior. CYP1A2, CYP2C19, and CYP2D6 presented larger bottleneck radii for tunnel 2c, indicating isoform-dependent behavior of the gating mechanisms, as described in the literature [15]. In CYP3A4, we could not detect any enlargement of these tunnels in response to a ligand bound to the H1 site.

The radius of gyration can be used to evaluate the compactness of a protein structure and thus to analyze the conformational state of protein [28,29]. To monitor the conformational state of the enzymes during these simulations and detect a potential shift towards an open conformation, we computed the radius of gyration for each replica (Figure S12). However, the values remained stable throughout all simulations, indicating no large deviations from the closed, compact conformation observed in crystal structures. Hence, this stands in contrast to the mentioned observations in CYP101A1 [14]. Potentially, such large conformational changes in mammalian membrane-anchored CYPs take place beyond the microsecond timescale [30]. Furthermore, the structured membrane lipids present in our simulations may impede certain conformational adaptations in the studied timescale, while the CYP101A1 is a soluble protein and was studied in an aqueous environment [14]. Evidence for the influence of biological membranes on protein conformations was provided for various proteins [31,32]. Thus, an allosteric modulation of the enzymes studied in the present work by ligand bound to the H1 site seemed to trigger dynamical conformation effects affecting only the tunnel microenvironment, with no apparent reach on the overall protein fold.

In a next step, we aimed to study the translocation of ligands through the identified pathways under the influence of a ligand bound to the H1 site and compared the results to systems with no such additional ligand. Generally, products of CYP-mediated metabolic transformations are more hydrophilic; thus, it was proposed that products might egress from solvent-exposed tunnels as opposed to substrates, which prefer tunnels directed toward the membrane to reach the binding pocket [10,15]. Thus, the selection process between access and egress tunnels is thought to be dictated by the physicochemical properties of the ligand. To avoid biasing the route selected by the ligand by placing it near an entrance at the outer surface of the enzyme, we decided to study the ligand dissociation from the binding pocket, which constitutes the inverse process based on the current rationale. Ligand unbinding is a comparatively slow process that requires substantial simulation times and represents a so-called rare molecular event. There are several methods available that enhance the sampling of such rare events by applying various biasing potentials on top of the regular force field. These techniques include steered MD, random-accelerated MD, umbrella sampling, protein energy landscape exploration, accelerated MD, as well as metadynamics simulations [9,26,33,34,35,36]. In the metadynamics protocol, Gaussian potentials are applied toward collective variables (CVs) defining the reaction coordinate of such a rare event [34]. Here, we selected the distance of the ligand center of mass to the heme iron atom within the active site as CV. We conducted twenty replica simulations per enzyme, with half of them having a ligand bound to the H1 site (Table 1). Except two simulations without allosteric ligand in CYP2C8, the ligands completely egressed from the binding pocket in all simulations. In analogy to the above-described cMD simulations, we compared the RMSF values of the metadynamics simulations to the ones obtained from the membrane equilibration (Figure 2C and Figure S13). Generally, the values were higher in the metadynamics simulations, especially if no allosteric ligand was bound to the H1 site. The most significant changes could be detected in the region of the F-G loop, as well as the C-terminus of helix B.

**Figure 3:**
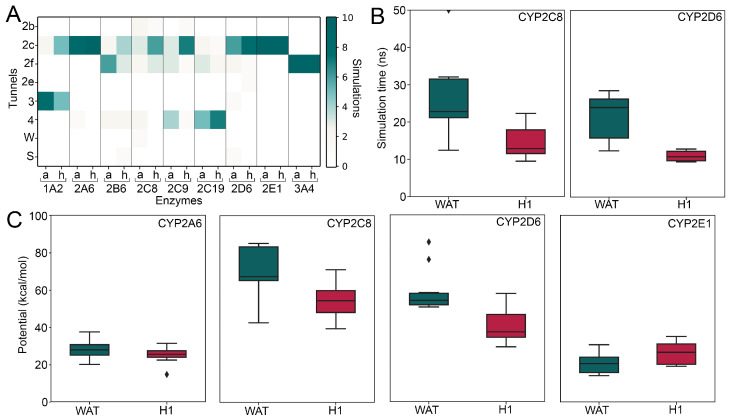
Results from metadynamics simulations. (**A**) Heat map of selected pathways with (h) and without (a) allosteric ligand. (**B**) Boxplots of simulation times (ΔT) until the ligand completely dissociated from the active site. (**C**) Boxplots of maximal potential (P_max_) registered until the ligand completely dissociated from the active site. ⧫: allosteric ligand.

To relate the results from the metadynamics simulations to the bottleneck radii obtained in the cMD simulations, we monitored the tunnels selected by the ligands to egress from the binding site (Figure 3A). In accordance to the bottleneck radii, we observed more trajectories during which the ligand selected tunnel 2c when an allosteric ligand was present in CYP1A2. In contrast to the cMD simulations, which indicated tunnel 2b to be enlarged if an allosteric ligand was bound to H1, the ligands preferred different tunnels in CYP2A6 and CYP2B6. In analogy to the results from the cMD simulations, there was a slight preference for tunnel 2f in CYP2C8 in response to H1 interactions. While there was no consensus between both techniques in the closely related CYP2C9 and CYP2C19, there was a preference for tunnel 2c as selected tunnel in CYP2D6 in the presence of an allosteric ligand in accordance with the observed bottleneck radii. We observed only a single pathway to be of relevance, independent of interactions with the H1 site in CYP2E1 and CYP3A4, indicating a missing functionality for the H1 site in these enzymes in addition to the above-mentioned small-molecule and organic probe densities.

Next, we computed the maximal biasing potential (P_max_) deposited during the dissociation, as well as the simulation time (ΔT) elapsed until the ligand completely dissociated (Table 2, Tables S4 and S5). Both parameters have been correlated with residence times of ligands in previous work [37,38] and, thus, they can be used to study the influence of the H1 interactions on ligand translocation. The significance of the differences was assessed using a two-sided t-test for both readouts. While only CYP2C8 and CYP2D6 presented significant improvements in ΔT or P_max_ in the presence of an allosteric ligand (Figure 3B, Figures S14 and S15), all enzymes besides CYP2C9, CYP2E1, and CYP3A4 presented lower values of the two metrics with H1 interactions. In accordance to all our previous results, the association of small molecules at the H1 seemed to have no effect on the functionality of ligand tunnels in CYP2E1 or CYP3A4, with the former even presenting more favorable values if there was no allosteric ligand present (Figure 3C).

## 3. Conclusions

Previous work on the prokaryotic CYP101A1 indicated an allosteric site involved in the regulation of ligand access to its buried binding pocket by shifting the enzyme toward an open conformation upon small-molecule interaction. Here, we conducted a multi-scale modeling approach to follow up on this hypothesis, focusing on the nine most relevant drug-metabolizing CYP enzymes in humans. Using MD simulations, we quantified the interaction of small molecules and organic solvents with this allosteric site, here denoted as H1 site, which is located among helices C, E, and H. These interactions indicated that all studied enzymes besides CYP2E1 and CYP3A4 have the potential to bind ligands at the H1 site, although the observed interactions in CYP2C19 were comparatively limited. When we analyzed the conservation of residues in the H1 site, we discovered the whole CYP2 family to share a high degree of similarity as opposed to CYP1A2 and CYP3A4. To potentially reproduce previous findings on CYP101A1, we restrained a ligand to the H1 site and analyzed the three most relevant tunnels, which may be used by ligands to access the active site, in a triplicate of microsecond MD simulations. In CYP2B6, CYP2C8, CYP2C9, and CYP2C19, we could observe an enlargement of tunnel 2f, which is separated from tunnel 2b by the F-G loop. The structural adaptation of this loop was shown to mediate the switch to an open conformation, and thus, an enlargement of either tunnel 2b as it took place in CYP2A6, CYP2B6, and CYP2E1, or tunnel 2f, points towards such an adaptation. Interestingly, all members of the CYP2 family, with the exception of CYP2D6, presented such a change in accordance with previous results. As in the previous analyses, we could not detect any enlargement of tunnels in CYP3A4. The diverging behavior of CYP3A4 in our analyses might originate from the high promiscuity of the enzyme compared to the remaining CYPs. Thus, different mechanisms controlling the conformational state of CYP3A4 might be involved. Despite the results regarding the changes of bottleneck radii, we could not detect any significant changes in the radius of gyration during the microsecond MD simulations. Hence, we could not detect a transition to an open conformation in our simulations. To study ligand translocation directly, we conducted metadynamics simulations by selecting the distance of the ligand to the heme iron atom as CV. Although it was only significant in CYP2C8 and CYP2D6, all studied enzymes except for CYP2C9, CYP2E1, and CYP3A4 presented either shorter residence in the active site or a lower maximal potential to induce ligand dissociation. Thus, interactions at the allosteric site indeed facilitated the translocation of ligands. In contrast, CYP2E1 presented a statistically significant increase in both maximal potential and simulation time for the ligand to dissociate. In conclusion, the results from different computational techniques suggested that the findings in CYP101A1 can be partially translated to several mammalian enzymes of the CYP2 family, even though no complete transition to an open conformation could be observed.

## 4. Materials and Methods

### 4.1. Bioinformatics Analysis

All protein sequence formats were obtained from the UniProt database [39] in FASTA format according to the accession codes listed in Table S7. Based on our previous work with CYP2D6 [8], we selected the 18 residues S137, T138, L139, R140, N141, L142, G143, L144, G145, K146, L149, L189, P268, R269, D270, L271, A274, and A277 to define the H1 allosteric site. Based on a multiple sequence alignment of the catalytic domains, we identified the corresponding residues in the remaining enzymes. For the sequence alignment, we selected the Clustal W algorithm [40] within the UGENE suite of tools (v34.0) [41]. The conservation was determined according to the amino acid groups provided in our previous work [42]. If a residue was in the same group, we assigned it a conservation value of 0.5, while a value of 1.0 was given for identical residues, and 0 was assigned if none of the above-mentioned conditions was fulfilled.

### 4.2. Model Building

The crystal structures of all studied CYPs were obtained from the Protein Data Bank [43] according to the accession codes provided in Table S7. For proteins deposited as multimers, only chain A was retained without organic solvents, histidine tags, or ions. From the obtained PDB files, FASTA sequences were extracted using an in-house Python routine. The generated sequences were aligned to the wild-type protein with the ClustalW algorithm [40] in the UGENE toolkit [41]. Mismatched amino acids were mutated to the ones corresponding to the wild-type enzyme in the 3D Builder panel within the Maestro Small-Molecule Drug Discovery suite (v2019-3) [44]. Next, the structures were processed with the Protein Preparation Wizard [45] by assigning bond orders, adding hydrogen atoms, predicting protonation states with Epik, and reorienting the hydrogen bonding network with PROPKA at pH 7.4. Next, the structures were subjected to a restrained minimization with the OPLS3e force field to a convergence threshold of 0.3 Å for protein heavy atoms. Missing residues and side chains were added using Prime. The heme moieties of the protein were modeled with six coordination partners to the Fe^3+^ ion including cysteine, the four porphyrin nitrogens, as well as an uncharged oxygen atom. If there was an inhibitor present in the active site, it was replaced with a structurally similar substrate that, however, is a substrate of the respective enzyme according to the review article by Rendic and colleagues [46]. These replacement and superposition procedures are detailed in Figure S16. To obtain systems covering the complete protein sequence, the transmembrane anchor was modeled as ideal alpha helix in the 3D Builder panel within Maestro by considering residues missing in the crystal structures. The anchors were placed perpendicular to the membrane plane along the z-axis and were aligned the C-terminus of the anchor to the N-terminus of the globular domain based on their backbone carbonyl atoms. Ultimately, we modeled a covalent bond linking the two parts. To avoid steric clashes, torsion angles of the membrane anchor residues were adjusted if it was necessary. A prequilibrated POPC membrane leaflet was placed on the predicted position by the Orientations of Proteins in Membranes (OPM) protocol accessible at the PPM server, which estimates the membrane position based on energetic contributions [47], as previously described [21]. All used ligand structures in this work were pre-processed using the LigPrep routine at a pH of 7.4 for ionizable functional groups and the OPLS3e force field to obtain energy-minimized conformers. Based on the Ligprep output, we retained the most favorable protomer for each ligand.

### 4.3. MD Simulations

All classical MD simulations were performed with the Desmond (v2019-1) simulation engine [48]. Five different sets of MD simulations were conducted: (i) simulations with 20 ligand molecules arbitrarily placed around the enzymes, (ii) cosolvent simulations, (iii) equilibration simulations of membrane-anchored CYPs, (iv) production simulations with allosteric ligand restrained to the protein surface, and (v) metadynamics simulations. All simulations were treated with the default equilibration protocol of Desmond before the production phase was conducted in an NPT ensemble at atmospheric pressure regulated by the Martyna–Tobias–Klein barostat and a temperature of 310 K maintained by the Nose–Hoover thermostat. The orthorhombic periodic boundary systems were solvated with TIP3P water molecules, counterions were used to neutralize the systems, and the OPLS_2005 force field was selected. In all simulations, the orthorhombic simulation box was defined with a distance cut-off of at least 10 Å to the nearest atom in all three cartesian directions. The time step of the RESPA integrator was set to 2 fs, long-range interactions were treated with the u-series algorithm [49], and bonds to hydrogen atoms controlled with M-SHAKE. For replica simulations, the random seed for initial velocities was modified.

For the first set of simulations, the membrane anchor was not included, and the cocrystallized ligands were removed from the prepared structures. For each CYP, 20 substrate molecules were randomly placed around the enzyme to monitor the association of drug-like ligands on the surface of the enzyme (Table S8). A duration of 500 ns per enzyme was selected with atomic coordinates recorded at an interval of 50 ps. Cosolvent MD simulations were conducted with the Mixed Solvent MD workflow within the Desmond (v2019-1) simulation engine [48]. As probe molecules, we selected isopropanol, acetonitrile, and pyridine at a concentration of 5% (by volume). Again, the orthosteric ligand was removed from the prepared protein structures without membrane anchor. The simulations were conducted with 10 replicas per cosolvent resulting in 30 individual simulations per enzyme. After an equilibration phase of 15 ns, the association of the probe molecules was sampled for 5 ns, leading to a total simulation time of 600 ns per system.

The third set of simulations was conducted on the complete protein-membrane systems in order to equilibrate them. The simulation time was set to 300 ns, and atomic coordinates were recorded at an interval of 30 ps.

The fourth set of simulations were started from the last MD frame of the prequilibrated protein-membrane systems. Using the rebuild_cms.py script that comes with Maestro, new systems were generated with a ligand bound to the H1 site. To obtain a starting conformation of the selected ligand bound to the H1 allosteric site, we used the Glide standard-precision docking protocol [50]. The selected ligands are given in Table S9 and Figure S17. We defined the search space for docking considering the position of acetaminophen bound to CYP2D6 H1 from our previous work. Water molecules overlapping with ligand atoms were removed assuming the probe size of 1.65 Å. After initial 5 ns of simulation to allow the ligand to freely accommodate within the allosteric site, we applied harmonic distance restraints with a force constant of 2.5 kcal·mol^−1^·Å^−1^ between the central atom of the ligand and α-carbons of three residues of the protein (Table S10) located in the comparatively rigid helices C, E, and I. These simulations were conducted in triplicate with a duration of 1 μs and atomic coordinates stored at an interval of 100 ps. The metadynamics simulations were also conducted with the Desmond simulation engine. The simulation systems were retained from the above-mentioned production simulations with a ligand restrained to the H1 site combined with an orthosteric ligand. As CV, we selected the distance between the centroid of the ligand and the heme iron atom with a wall of 45 Å. We retained the height of the Gaussian at 0.03 kcal/mol as well as the width of 0.05 Å according to the default specification.

### 4.4. Evaluation of the MD Trajectories

For all simulations, except for the comparatively short cosolvent MD simulations, we computed RMSD and RMSF values using the Simulation Interaction Diagram panel in Maestro. For the metadynamics simulation, we truncated the trajectories beforehand to only represent the dissociation process using the trajectory_extract_subsystem.py Python routine that comes with Maestro.

Initially, the first set of simulations was conducted to obtain a stable pose of the ligands bound to the H1 site that could be superimposed to the prequilibrated membrane models for further procedures. However, conformational changes of the protein surface prevented this procedure, as they would have introduced large steric clashes. Thus, we evaluated these simulations to obtain additional insight into the preference of small-molecule association on the enzyme surface. We used an in-house Python routine computing the cumulative number of ligand heavy atoms within 5 Å distance to protein α-carbons for each MD frame and normalized this count to the total number of ligand heavy atoms.

To validate our membrane models with experimental parameters, we determined the heme tilt angle as well as the burying depth of the membrane-anchored proteins during our equilibration simulations. The heme tilt angle was defined as the angle between the heme plane defined by the porphyrin nitrogens and the membrane normal (z-axis) [11,21]. The burying depth was defined as distance between the mass center of the protein considering α-carbons and the centroid of the POPC C1-carbons [21,51]. Both of these validation parameters were determined for every frame of the trajectories in analogy to our previous work [8].

Tunnels connecting the buried active site of CYPs to the surrounding solvent environment were computed using CAVER (v3.0) [27] for the microsecond simulations of the full-length proteins embedded in a membrane. The starting point for the tunnel computation was determined in CAVER Analyst (v1.0) [52] by selecting the heme, as well as two additional residues in the binding site based on a structural alignment to CYP2D6, for which we defined suitable residues in our previous work (Table S11). Similarly, as reported in our previous analyses on CYP2D6 [8,21], we selected a clustering threshold of 4.5 for the computation. As we observed four simulations presenting a dissociation of the allosteric ligand during the preequilibration of 5 ns without restraints, we discarded these trajectories from the tunnel computation (Table S12). The radius of gyration was determined from MD frames according to the publication of Lobanov and colleagues [29].

While we visually determined the simulation time ΔT when the ligand completely dissociated from the protein in the metadynamics simulations, we derived the maximal potential P_max_ during the egress process using the metadynminer toolkit based on R scripting language [53]. The statistical significance of the average simulation time until ligand egress, the maximal potentials, as well as the bottleneck radii was evaluated using ttest_ind_from_stats routine that comes with the Python-SciPy module at the *p* = 0.1 significance level.

## Figures and Tables

**Table 1:** Simulations conducted in this work.

Type	Membrane	Duration (ns)	Replicas ^a^	Lig_Ortho_^b^	Lig_Allo_^b^
Cosolvent	no	60	10	no	no
Association	no	500	1	no	yes ^c^
Equilibration	yes	300	1	yes	no
Sampling	yes	1005	3	no	yes
Metadynamics	yes	50	10	yes	no
Metadynamics	yes	50	10	yes	yes

^a^ Number of replicas per enzyme. ^b^ Indicator if a ligand was present at the respective site. ^c^ Transient presence of ligand.

**Table 2:** Statistical analysis of metadynamics simulations.

Enzyme	Outcome ΔT ^a^	Outcome P_max_ ^a^	Significance	2b	r_B_ ^b^ 2c	2f
CYP1A2	H1 (0.386)	H1 (0.400)	no	=	+	=
CYP2A6	H1 (0.237)	H1 (0.179)	no	+	=	=
CYP2B6	H1 (0.204)	H1 (0.522)	no	+	n/a ^c^	+
CYP2C8	H1 (0.010)	H1 (0.025)	yes	–	–	+
CYP2C9	WAT (0.675)	WAT (0.559)	no	=	–	+
CYP2C19	H1 (0.454)	H1 (0.862)	no	–	+	+
CYP2D6	H1 (0.001)	H1 (0.001)	yes	–	+	–
CYP2E1	WAT (0.232)	WAT (0.085)	yes	+	–	=
CYP3A4	H1 (0.571)	WAT (0.713)	no	n/a ^c^	=	–

^a^ Outcome (H1 for allosteric, WAT for no allosteric) of the metadynamics simulations for the simulation time (ΔT) and maximal potential (P_max_). ^b^ Statistical outcome of potential changes in bottleneck radius (r_B_). ^c^ Tunnel not present.

## Data Availability

Additional data including MD trajectories and other readouts are available upon written request to the authors.

## Supplementary Materials

The following are available online at https://www.mdpi.com/article/10.3390/ijms222413215/s1.
