# Supplementary material for: A Conserved Allosteric Site on Drug-Metabolizing CYPs: A Systematic Computational Assessment
Source: Int J Mol Sci. 2021 Dec 8;22(24):13215. doi: 10.3390/ijms222413215 (PMC8707821; doi:10.3390/ijms222413215)
Supplement: Supplementary file 1 [file ijms-22-13215-s001.zip › ijms-1396374-supplementary.pdf]

Supporting Information: Allosteric Site on Drug-Metabolizing CYPs Relevant For Ligand Kinetics: A Systematic Computational Assessment

André Fischer and Martin Smieško*

Computational Pharmacy, Departement of Pharmaceutical Sciences, University of Basel, Klingelbergstrasse 61, 4056 Basel, Switzerland

E-mail: martin.smiesko@unibas.ch

Abstract

Cytochrome P450 enzymes (CYPs) are the largest group of enzymes involved in human drug metabolism. Ligand tunnels connect their active site buried at the core of the membrane-anchored protein to the surrounding solvent environment. Recently, evidence of a superficial allosteric site, here denoted as hotspot 1 (H1), involved in the regulation of ligand access in a soluble prokaryotic CYP emerged. Here, we applied multi-scale computational modeling techniques to study the conservation and functionality of this allosteric site in the nine most relevant mammalian CYPs responsible for approximately 70% of drug metabolism. In total, we systematically analyzed over 44 μs of trajectories from conventional MD, cosolvent MD, and metadynamics simulations. Our bioinformatics analysis and simulations with organic probe molecules revealed the site to be well conserved in the CYP2 family with the exception of CYP2E1. In the presence of a ligand bound to the H1 site, we could observe an enlargement of a

ligand tunnel in several members of the CYP2 family. Further, we could detect the facilitation of ligand translocation by H1 interactions with statistical significance in CYP2C8 and CYP2D6, even though all other enzymes except for CYP2C19, CYP2E1, and CYP3A4 presented a similar trend. As the detailed comprehension of ligand access and egress phenomena remains one of the most relevant challenges in the field, this work contributes to its elucidation, and ultimately, helps in estimating the selectivity of metabolic transformations using computational techniques.

Supporting Introduction

Table S1: Enzyme overview.

**Enzyme**	**Relevance**[a]	**Substrate characteristics**	**Example substrates**
CYP1A2	8.9%	planar, aromatic	Caffeine, Lidocaine
CYP2A6	3.4%	non-planar, low molecular weight	Nicotine, Coumarin
CYP2B6	7.2%	non-planar, lipophilic, neurtal or weakly basic	Buproprion, Efavirenz
CYP2C8	4.7%	large, weakly acidic	Amodiaquine, Palcitaxel
CYP2C9	12.8%	weakly acidic, with hydrogen bond acceptor	Diclofenac, Warfarin
CYP2C19	6.8%	neutral or weakly basic	Omeprazole, Imipramine
CYP2D6	20.0%	basic with protonatable nitrogen	Codeine, Oxycodone
CYP2E1	3.0%	small, neutral hydrophilic, planar	Chlorzoxazone, Paracetamol
CYP3A4	30.2%	large, lipophilic	Atorvastatin, Midazolam

The relevance (fraction of clinically used drugs metabolized by respective enzyme), characteristics of typical substrates, and well-known example substrates of the CYPs studied in this work. This table was adapted from the work of Zanger and colleagues.[S1]

Supporting Results and Discussion

Simulation techniques and model validation

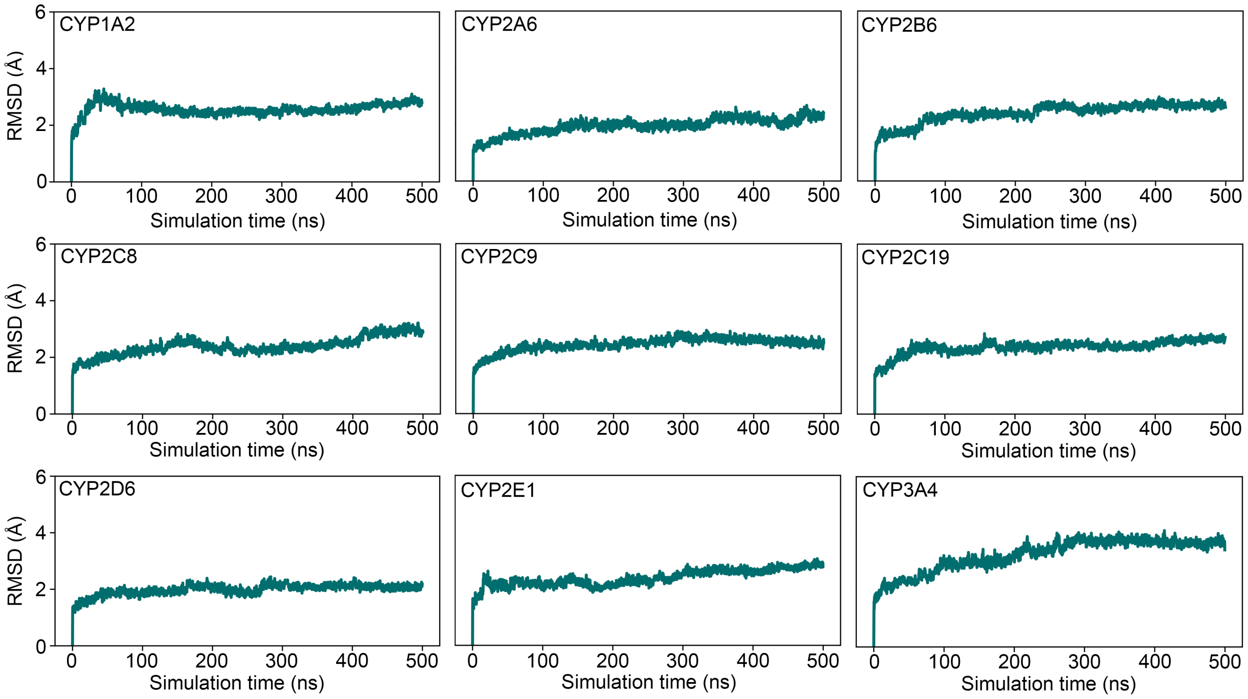

Figure S1: RMSD of association simulations.

Table S2: Membrane model validation.

**Enzyme**	**Heme tilt angle (°)**	**Burying depth (Å)**
CYP1A2	66.7 ± 3.7	36.0 ± 1.1
CYP2A6	39.1 ± 3.8	38.5 ± 1.7
CYP2B6	66.3 ± 3.4	38.9 ± 1.6
CYP2C8	63.8 ± 3.5	38.6 ± 1.4
CYP2C9	68.6 ± 3.4	37.3 ± 1.3
CYP2C19	62.5 ± 3.7	36.9 ± 1.9
CYP2D6	55.3 ± 3.5	38.1 ± 1.3
CYP2E1	53.1 ± 3.9	38.4 ± 1.6
CYP3A4	75.3 ± 3.0	35.7 ± 1.0

Average values are given with standard deviation.

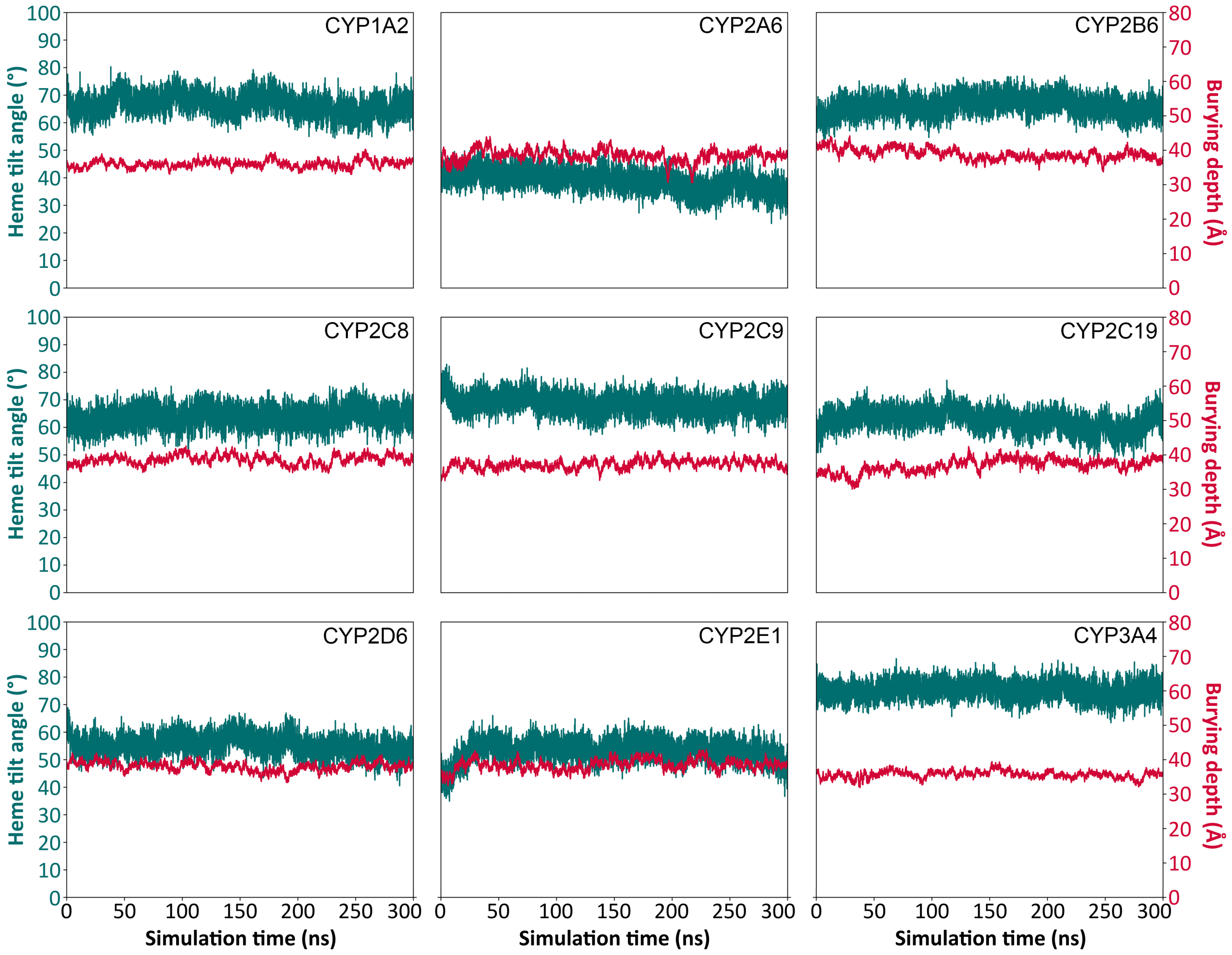

Figure S2: Time-evolved values of heme tilt angle (pine green) and buyring depth (red) during association simulations.

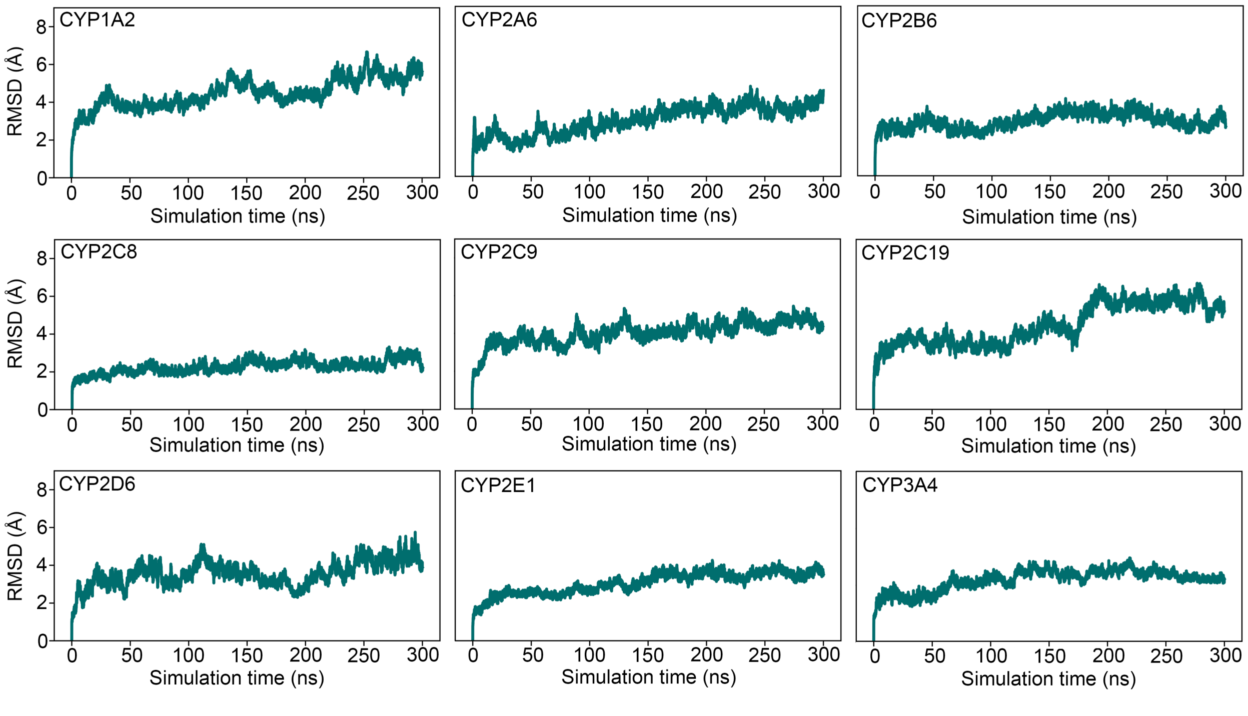

Figure S3: RMSD of membrane equilibration simulations.

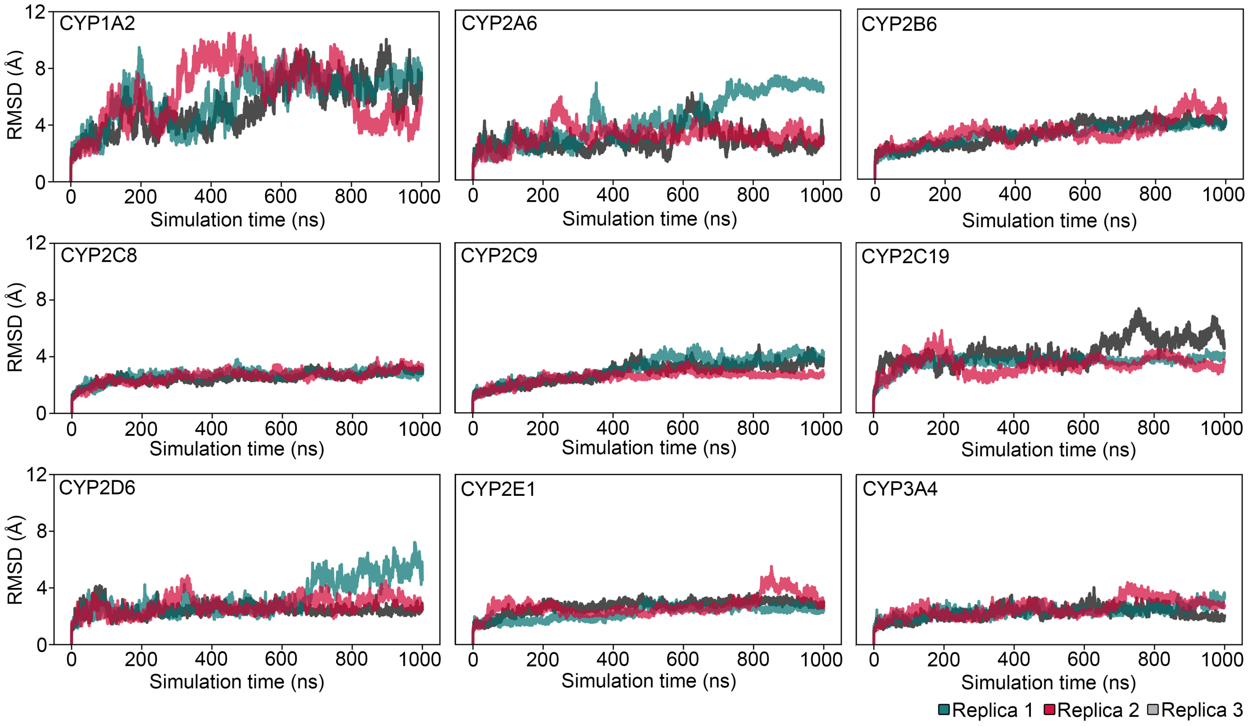

Figure S4: RMSD of sampling simulations.

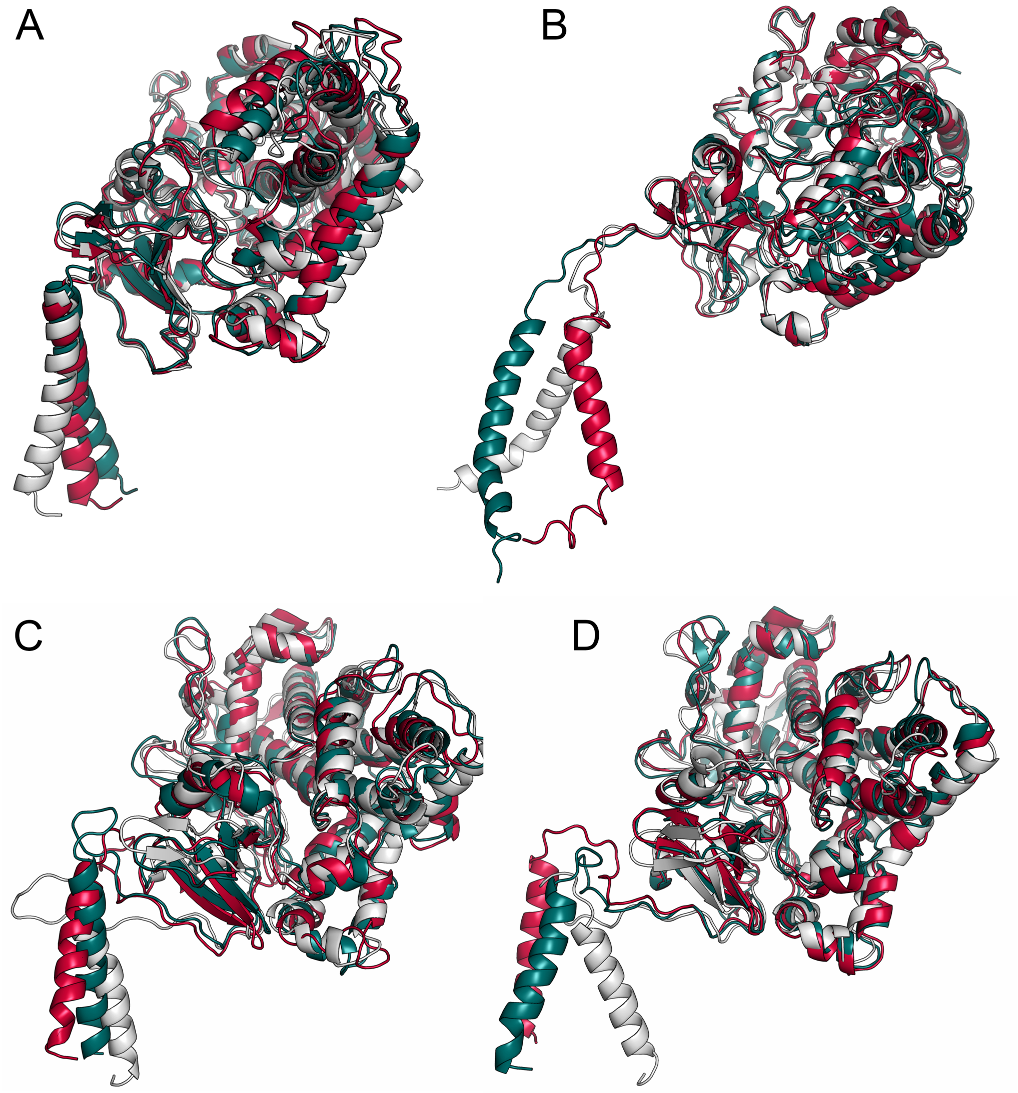

Figure S5: Depiction of conformational changes in sampling simulations for (A) CYP2C8, (B) CYP1A2, (C) CYP2C19, and (D) CYP2C19. The three replica simulations of each system are shown in different colors.

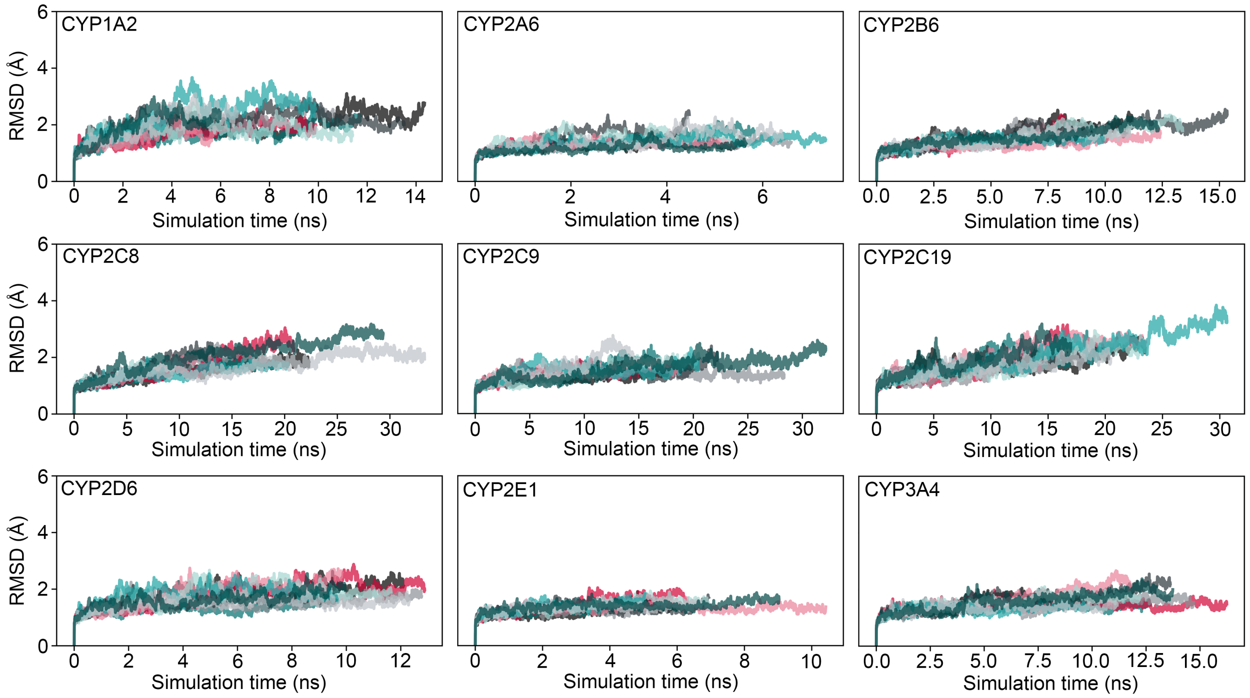

Figure S6: RMSD of metadynamics simulations (with allosteric ligand) in all enzymes studies here. Different replica simulations are indicated by different colors.

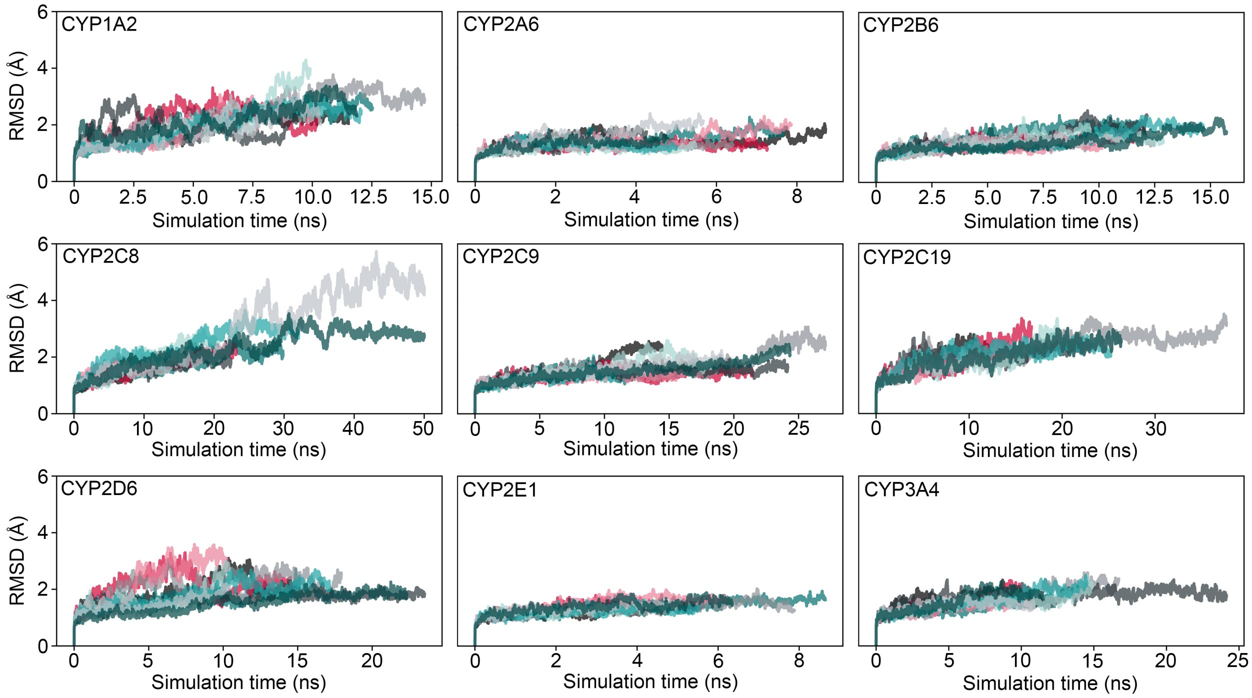

Figure S7: RMSD of metadynamics simulations (without allosteric ligand) in all enzymes studies here. Different replica simulations are indicated by different colors.

The effect of allosteric ligands bound to the H1 site is isoform-dependent.

Table S3: Statistics of bottleneck radii.

Enzyme	Lig_{allo}	Tunnel	n	Mean	SD
CYP1A2	no	2b	265	1.033	0.108
CYP1A2	yes		224	1.023	0.120
CYP1A2	no	2c	396	1.025	0.089
CYP1A2	yes		625	1.063	0.141
CYP1A2	no	2f	508	1.141	0.123
CYP1A2	yes		773	1.129	0.141
CYP2A6	no	2b	70	0.941	0.039
CYP2A6	yes		242	0.967	0.071
CYP2A6	no	2c	97	0.969	0.069
CYP2A6	yes		327	0.955	0.059
CYP2A6	no	2f	15	0.920	0.015
CYP2A6	yes		35	0.939	0.035
CYP2B6	no	2b	535	1.181	0.176
CYP2B6	yes		892	1.309	0.290
CYP2B6	no	2f	126	1.038	0.104
CYP2B6	yes		346	1.074	0.119
CYP2C8	no	2b	555	1.641	0.209
CYP2C8	yes		1455	1.468	0.274
CYP2C8	no	2c	555	1.605	0.262
CYP2C8	yes		1262	1.395	0.308
CYP2C8	no	2f	11	0.953	0.064
CYP2C8	yes		1154	1.326	0.253
CYP2C9	no	2b	5	0.982	0.060
CYP2C9	yes		526	1.118	0.145
CYP2C9	no	2c	556	1.512	0.215
CYP2C9	yes		370	1.055	0.131
CYP2C9	no	2f	62	0.959	0.044
CYP2C9	yes		222	0.973	0.068

Table S4: Statistics of bottleneck radii (continued).

Enzyme	Lig_{allo}	Tunnel	n	Mean	SD
CYP2C19	no	2b	465	1.342	0.240
CYP2C19	yes		133	1.109	0.251
CYP2C19	no	2c	76	1.178	0.338
CYP2C19	yes		505	1.510	0.306
CYP2C19	no	2f	238	1.093	0.227
CYP2C19	yes		839	1.609	0.477
CYP2D6	no	2b	354	1.289	0.244
CYP2D6	yes		1342	1.214	0.189
CYP2D6	no	2c	119	1.007	0.141
CYP2D6	yes		570	1.046	0.139
CYP2D6	no	2f	344	1.328	0.242
CYP2D6	yes		1119	1.192	0.178
CYP2E1	no	2b	302	1.054	0.153
CYP2E1	yes		333	1.087	0.156
CYP2E1	no	2c	556	1.640	0.203
CYP2E1	yes		498	1.454	0.295
CYP2E1	no	2f	61	0.956	0.059
CYP2E1	yes		35	0.962	0.062
CYP3A4	no	2c	38	0.983	0.085
CYP3A4	yes		34	1.054	0.331
CYP3A4	no	2f	555	2.161	0.261
CYP3A4	yes		1433	1.78	0.496

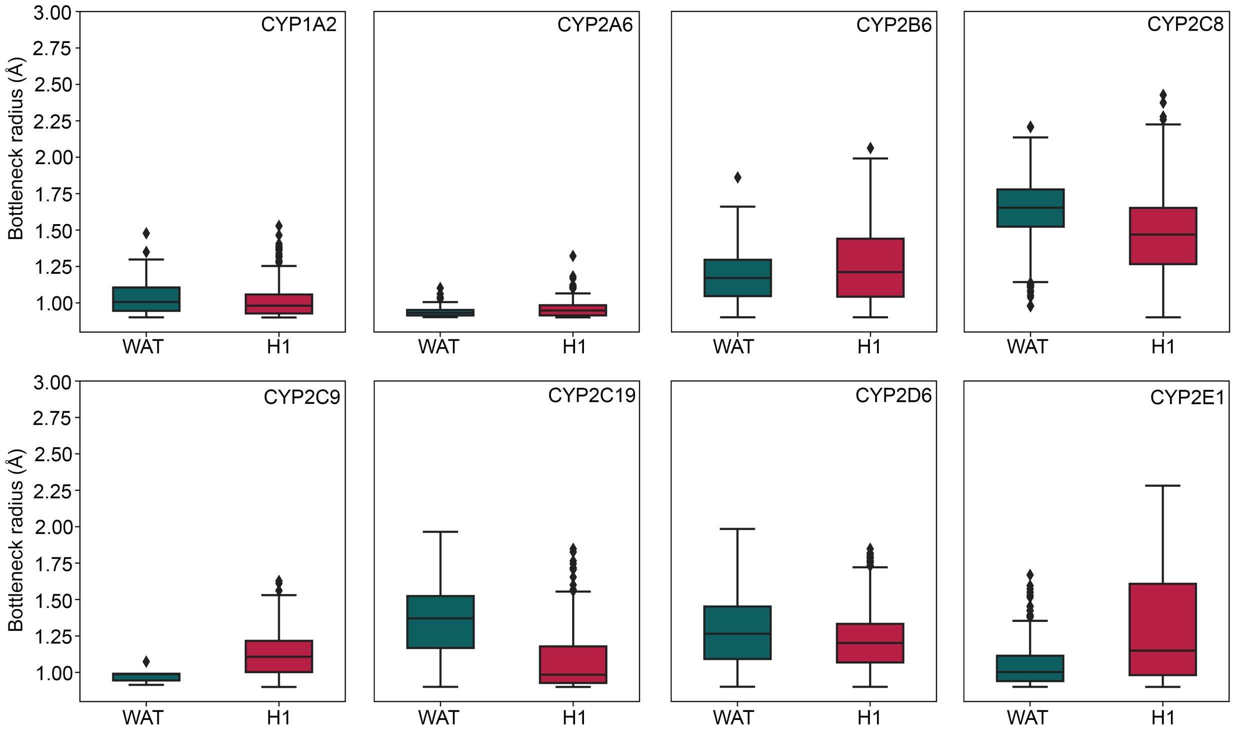

Figure S8: Boxplots of bottleneck radii for tunnel 2b. While ”WAT” indicates no allosteric ligand present, ”H1” indicates if a ligand was bound to H1.

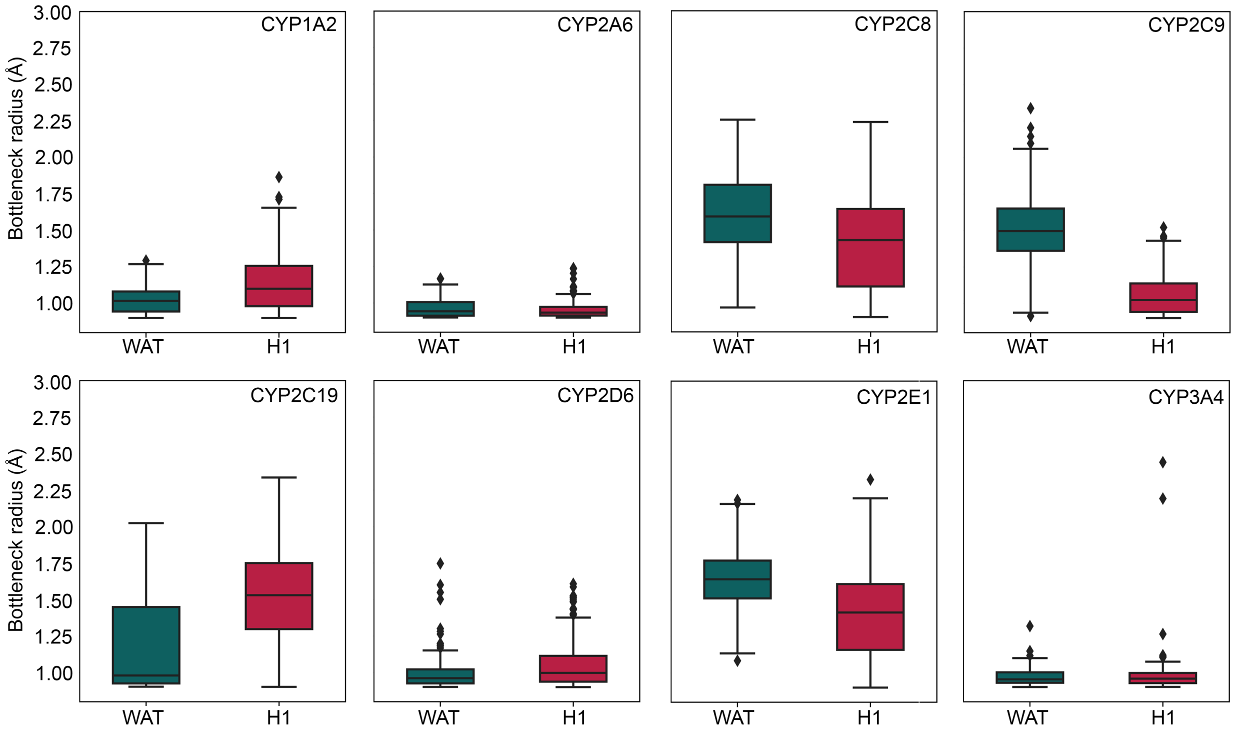

Figure S9: Boxplots of bottleneck radii for tunnel 2c. While "WAT" indicates no allosteric ligand present, "H1" indicates if a ligand was bound to H1.

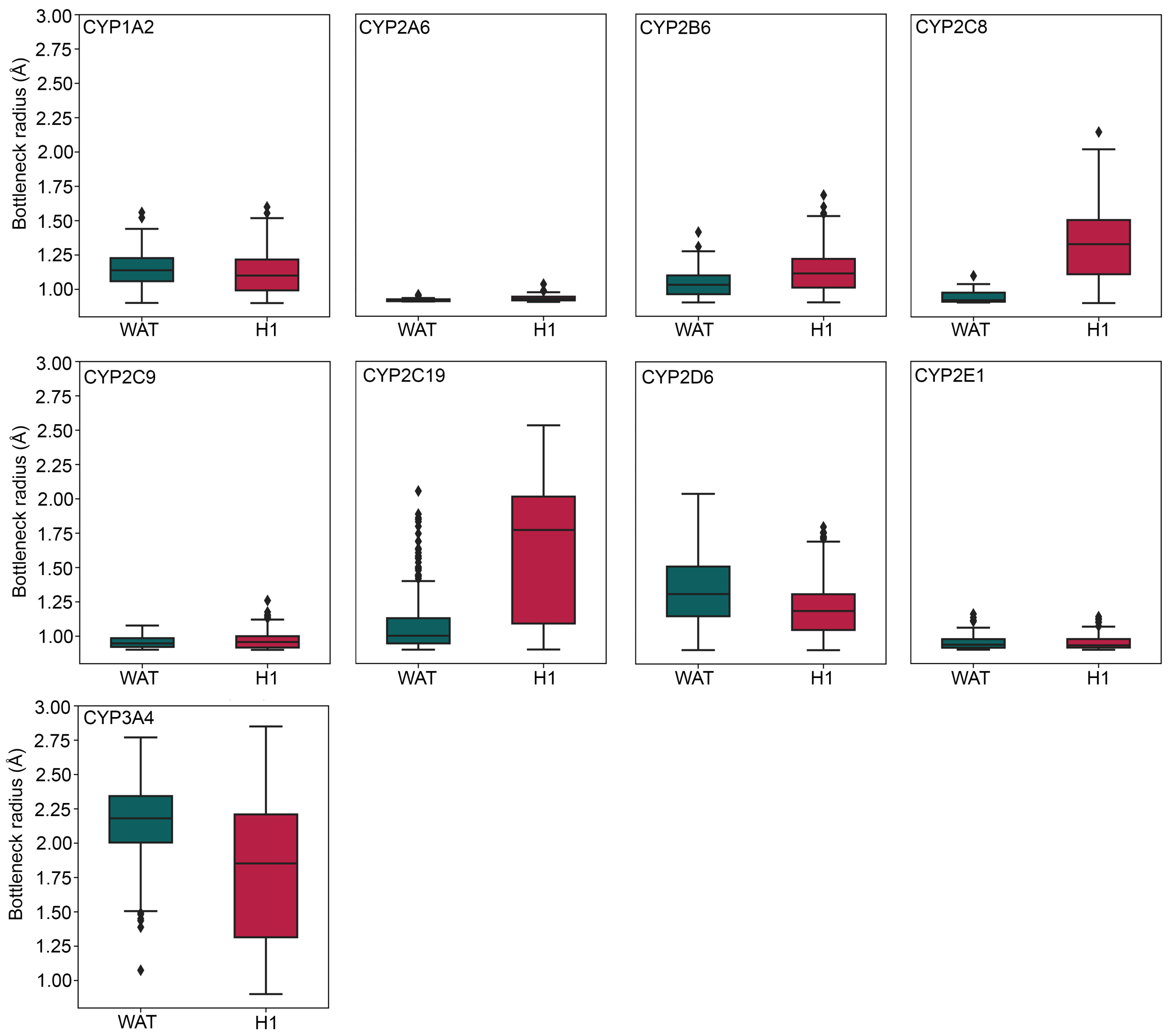

Figure S10: Boxplots of bottleneck radii for tunnel 2f. While "WAT" indicates no allosteric ligand present, "H1" indicates if a ligand was bound to H1.

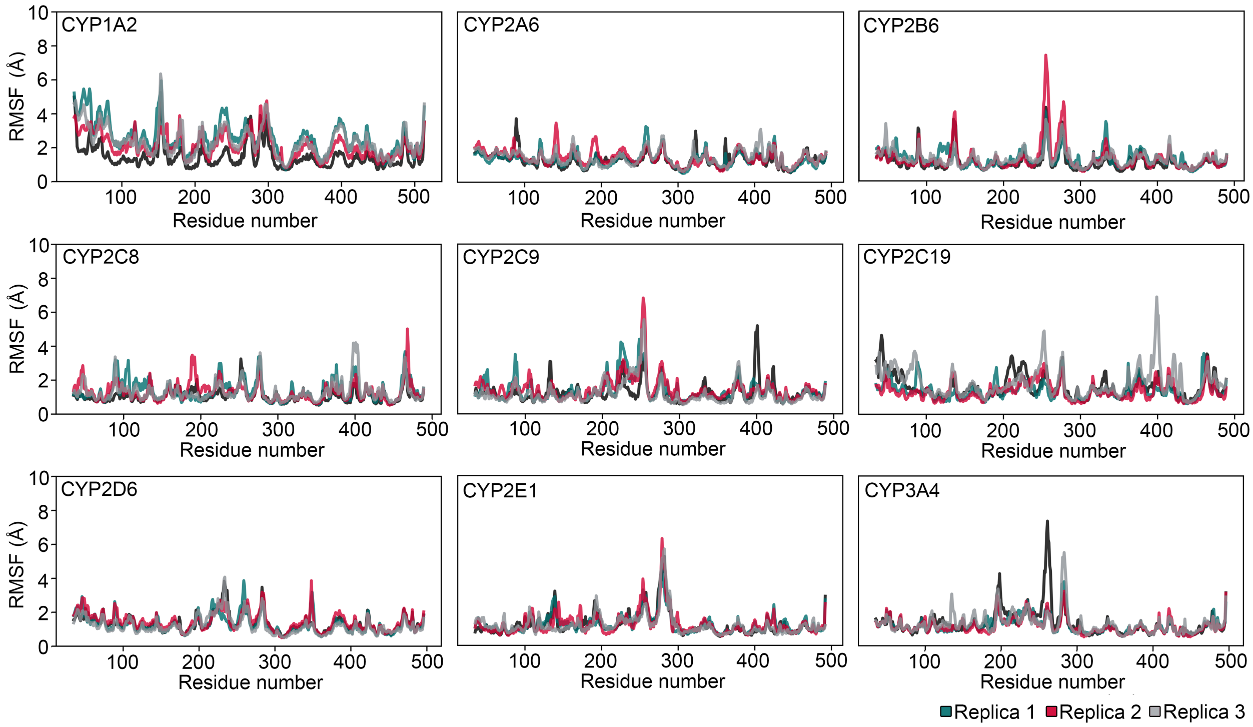

Figure S11: RMSF of sampling simulations for all studied enzymes.

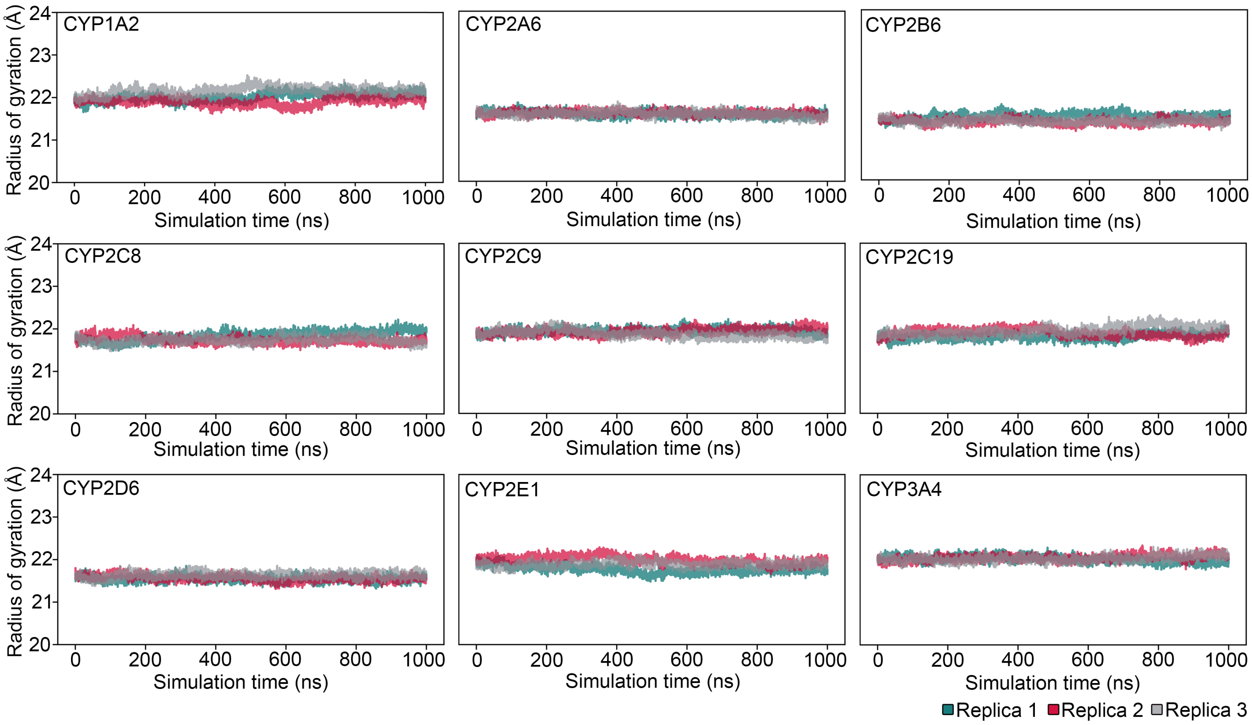

Figure S12: Radius of gyration during sampling simulations.

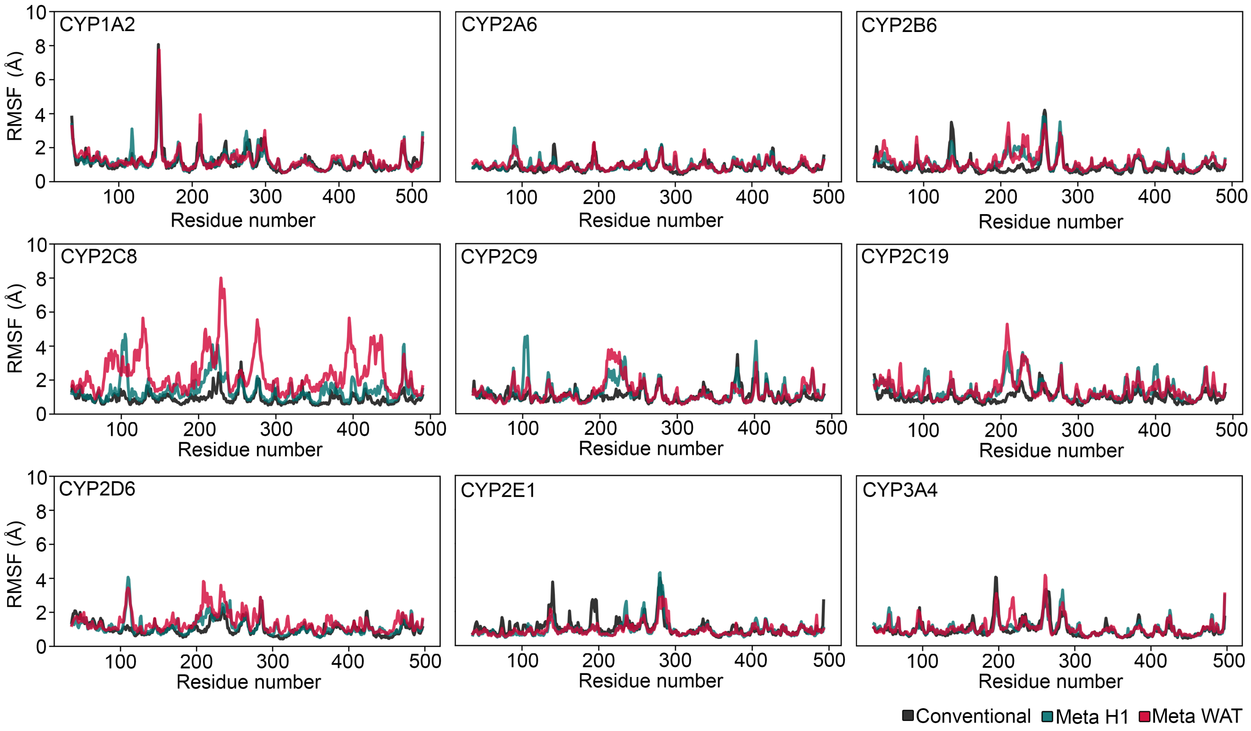

Figure S13: RMSF of metadynamics simulations compared to the ones obtained from conventional MD.

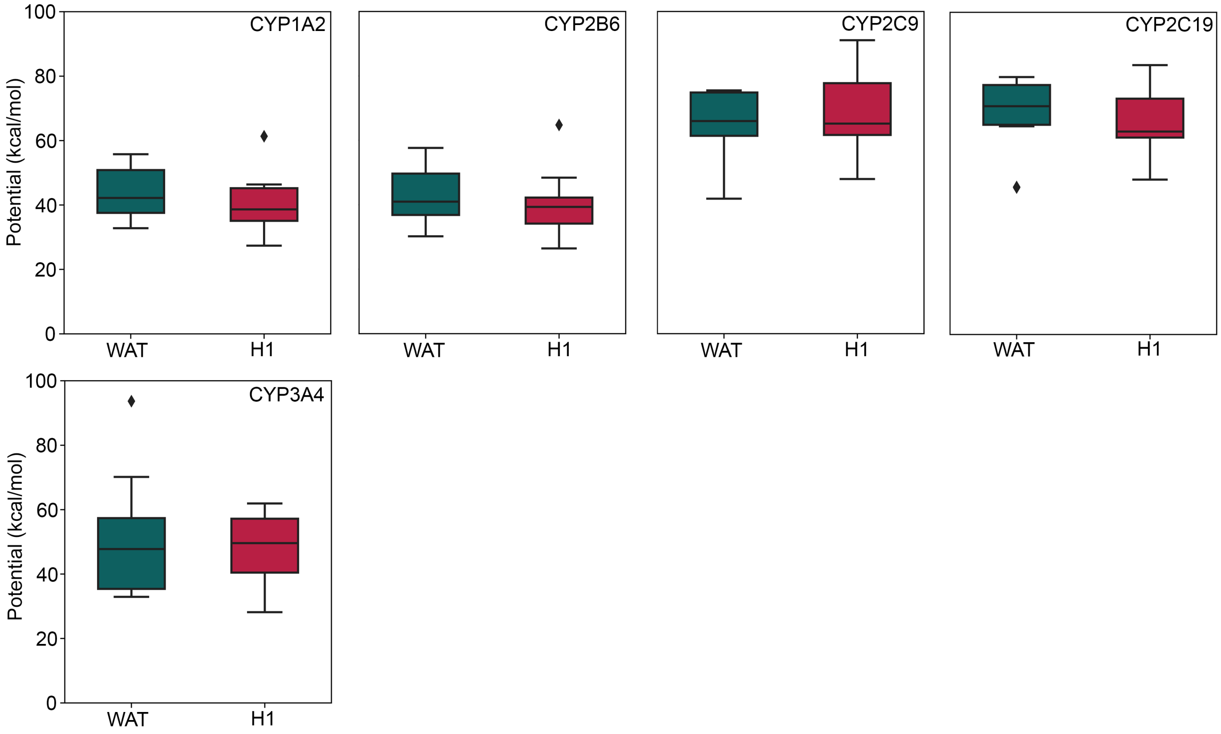

Figure S14: Boxplots of maximal potential (P_{max}) registered during metadynamics simulations.

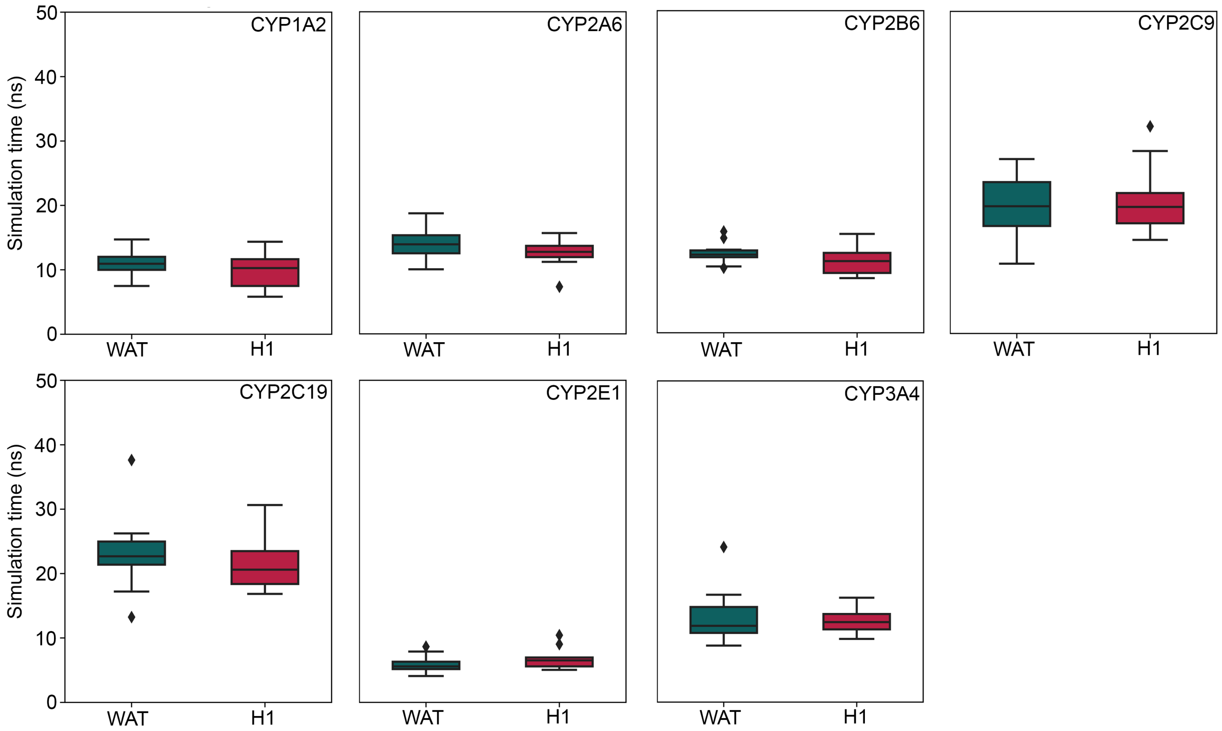

Figure S15: Boxplots of simulations times (ΔT) registered during metadynamics simulations.

Table S5: Statistics of maximal biasing potential.

Enzyme	Lig_{allo}	Tunnel	n	P_{max} (kcal/mol) Mean	SD	ΔT (ns) Mean	SD
CYP1A2	yes	2c	5	42.794	12.920	10.659	2.790
CYP1A2	no		2	44.104	10.188	10.883	0.909
CYP1A2	yes	3	5	38.676	4.175	9.329	3.376
CYP1A2	no		8	44.105	8.363	11.036	2.228
CYP1A2	yes	all	10	40.735	9.308	9.994	3.003
CYP1A2	no		10	44.105	8.119	11.005	1.989
CYP2A6	yes	2c	10	25.121	4.569	5.547	1.302
CYP2A6	no		9	27.076	3.984	6.051	1.327
CYP2A6	yes	all	10	25.121	4.569	5.898	1.331
CYP2A6	no		10	28.111	4.982	6.318	1.509
CYP2B6	yes	2c	4	34.652	5.453	9.780	0.957
CYP2B6	no		2	36.933	4.627	10.190	0.148
CYP2B6	yes	2f	3	44.176	3.700	12.720	0.590
CYP2B6	no		6	46.432	10.666	13.232	1.616
CYP2B6	yes	4	2	51.483	18.815	13.258	2.952
CYP2B6	no		2	38.937	3.959	12.083	0.541
CYP2B6	yes	all	10	40.056	10.977	11.228	2.185
CYP2B6	no		10	43.033	9.329	12.394	1.748
CYP2C8	yes	2c	6	48.788	5.873	12.398	2.617
CYP2C8	no		3	52.085	11.539	19.040	11.293
CYP2C8	yes	2f	3	63.399	6.637	20.468	2.088
CYP2C8	no		2	71.964	8.305	22.793	0.541
CYP2C8	yes	all	10	54.716	9.358	14.729	4.532
CYP2C8	no		10	75.437	25.199	27.575	13.362
CYP2C9	yes	2c	7	67.903	12.373	19.571	4.760
CYP2C9	no		3	61.515	13.990	16.127	5.315
CYP2C9	yes	all	10	67.941	14.328	20.658	5.681
CYP2C9	no		10	64.413	12.068	19.642	4.950

Table S6: Statistics of maximal biasing potential (continued).

				P_{max} (kcal/mol)		ΔT (ns)	
**Enzyme**	**Lig_{allo}**	**Tunnel**	**n**	**Mean**	**SD**	**Mean**	**SD**
CYP2C19	yes	2f	2	88.222	34.742	25.560	7.184
CYP2C19	no		3	77.834	21.233	27.447	8.862
CYP2C19	yes	4	7	62.884	12.778	19.848	2.837
CYP2C19	no		5	68.267	13.106	22.229	3.391
CYP2C19	yes	all	10	68.657	18.818	21.355	4.160
CYP2C19	no		10	70.053	16.574	23.196	6.370
CYP2D6	yes	2c	8	39.114	9.468	11.098	1.452
CYP2D6	no		6	56.748	9.729	19.781	6.570
CYP2D6	yes	all	10	40.332	8.952	11.033	1.433
CYP2D6	no		10	59.120	11.906	21.618	5.997
CYP2E1	yes	all	10	25.989	5.955	6.815	1.723
CYP2E1	no		10	21.144	5.937	5.943	1.411
CYP3A4	yes	all	10	48.443	11.299	12.625	2.010
CYP3A4	no		10	51.072	19.207	13.513	4.434

Materials and Methods

Model building

Table S7: Structure overview.

Enzyme	PDB ID	UniProt ID	Mutations to obtain wild-type
CYP1A2	2HI4	P05177	none
CYP2A6	1Z10	P11509	none
CYP2B6	5UAP	P20813	D28G, R29K, Y226H, K262R
CYP2C8	2NNI	P10632	none
CYP2C9	1OG5	P11712	K206E, I215V, C216Y, S220P, P221A, I222L, I223L, G296K
CYP2C19	4GQS	P33261	V490I
CYP2D6	3TDA	P10635	A31G, R32K, Y33L
CYP2E1	3GPH	P05181	N31K
CYP3A4	5TE8	P08684	L22A

Accession codes for Protein DataBank and UniProt database given for all enzymes along with the amino acid mutations to obtain the wild-type sequence.

Table S8: Substrates distributed around the enzymes.

**Enzyme**	**Residues**
CYP1A2	16x acetaminophen, 4x caffeine
CYP2A6	15x acetaminophen, 5x nicotine
CYP2B6	3x quinoline, 10x propofol, 7x nicotine
CYP2C8	10x ibuprofen, 5x propofol, 5x nicotine
CYP2C9	15x acetaminophen, 5x ibuprofen
CYP2C19	15x phenacetin, 5x nicotine
CYP2D6	20x acetaminophen
CYP2E1	15x acetaminophen, 2x phenacetin, 3x chlorzoxazone
CYP3A4	16x acetaminophen, 4x chlorzoxazone

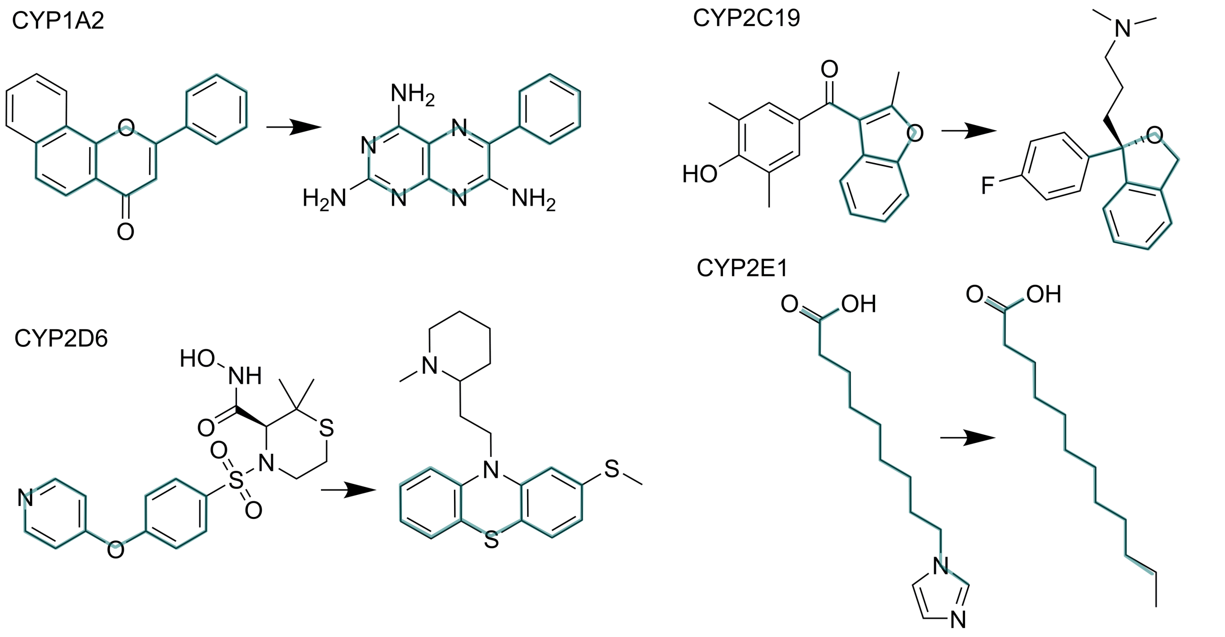

Figure S16: Ligands that were manually replaced by superposition during model building procedures based on the highlighted common scaffolds, which are depicted in pine green.

Table S9: Ligands studied in the metadynamics simulations.

**Enzyme**	**Allosteric**	**Orthosteric**
CYP1A2	acetaminophen	triamterene
CYP2A6	acetaminophen	coumarin
CYP2B6	acetaminophen	ZINC49942680
CYP2C8	ibuprofen	montelukast
CYP2C9	acetaminophen	(S)-warfarin
CYP2C19	phenacetin	60122187[a]
CYP2D6	acetaminophen	thioridazine
CYP2E1	acetaminophen	undecanoic acid
CYP3A4	acetaminophen	midazolam

[a] PubChem identifier (compound ID) given.

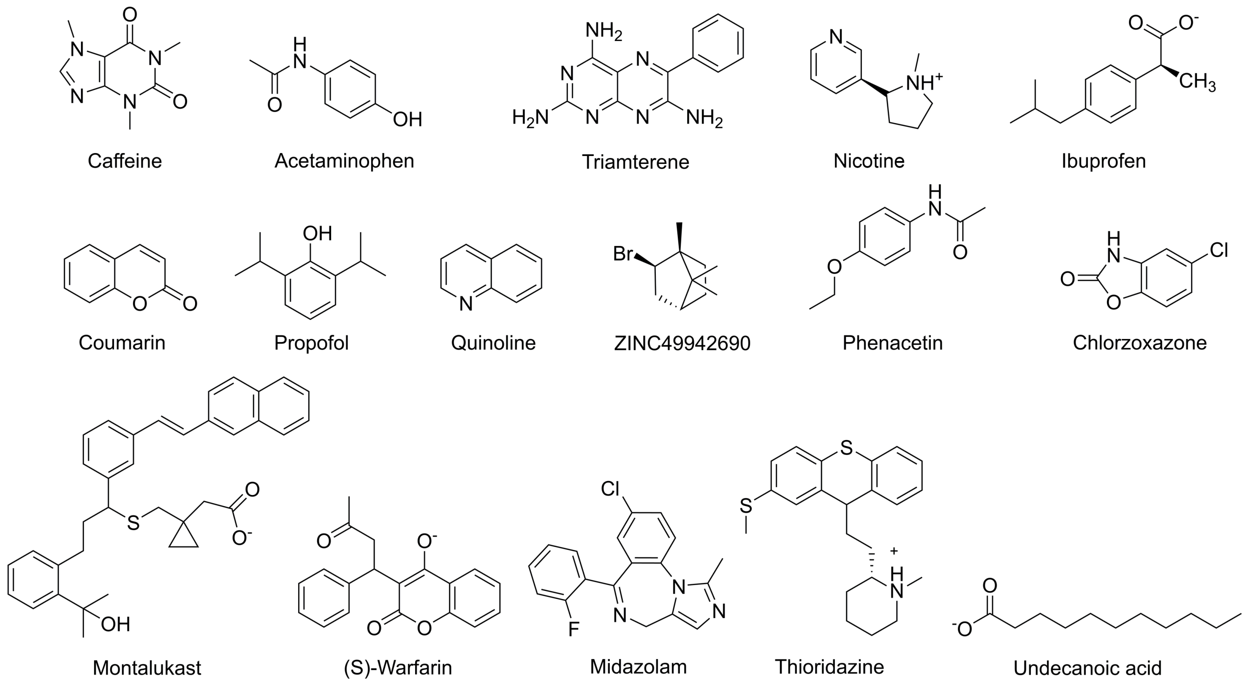

Figure S17: Two-dimensional structures of ligands studied in this work.

MD simulations

Table S10: Residues for harmonic distance restraints.

Enzyme	Helix C	Helix E	Helix I
CYP1A2	A140	V199	V311
CYP2A6	S131	V181	T295
CYP2B6	S128	I178	T292
CYP2C8	S127	V177	V291
CYP2C9	S127	V177	A291
CYP2C19	S127	V177	A291
CYP2D6	S135	V185	V299
CYP2E1	S129	V179	V293
CYP3A4	L133	V183	I300

Evaluation of the MD trajectories

Table S11: Residues for tunnel computation starting points.

Enzyme	Residues
CYP1A2	A230, D313
CYP2A6	T212, N297
CYP2B6	I209, S294
CYP2C8	L208, L294
CYP2C9	L233, D293
CYP2C19	V208, D293
CYP2D6	E216, D301
CYP2E1	L210, D295
CYP3A4	L216, I301

Table S12: Occupancy of H1 site during free MD.

Enzyme	Replica 1	Replica 2	Replica 3
CYP1A2	yes	no	yes
CYP2A6	yes	yes	yes
CYP2B6	yes	yes	no
CYP2C8	yes	yes	yes
CYP2C9	yes	yes	yes
CYP2C19	yes	yes	yes
CYP2D6	yes	yes	yes
CYP2E1	no	yes	no
CYP3A4	yes	yes	yes

References

(S1) Zanger, U. M.; Schwab, M. Cytochrome P450 enzymes in drug metabolism: Regulation of gene expression, enzyme activities, and impact of genetic variation. *Pharmacology and Therapeutics* **2013**, *138*, 103–141.
